# Tribological Behavior of AlCrSiN-Coated Tool Steel K340 Versus Popular Tool Steel Grades

**DOI:** 10.3390/ma13214895

**Published:** 2020-10-31

**Authors:** Kazimierz Drozd, Mariusz Walczak, Mirosław Szala, Kamil Gancarczyk

**Affiliations:** 1 Department of Materials Engineering, Faculty of Mechanical Engineering, Lublin University of Technology, 20-618 Lublin, Poland; k.drozd@pollub.pl (K.D.); m.walczak@pollub.pl (M.W.); 2 Department of Materials Science, Faculty of Mechanical Engineering and Aeronautics, Rzeszow University of Technology, Powstancow Warszawy 12, 35-959 Rzeszow, Poland; KamilGancarczyk@prz.edu.pl

**Keywords:** cold/hot-work steel, sliding, friction, wear testing, XRD analysis, wear mechanism, hardness, heat treatment, thin film, abrasion

## Abstract

The tribological performance of metalwork steel tools is of vital importance in both cold and hot working processes. One solution for improving metal tool life is the application of coatings. This paper investigates the differences in quantitative wear behavior and wear mechanisms between AlCrSiN-coated and bare steel K340 and five reference tool steels: X155CrVMo12-1, X37CrMoV5-1, X40CrMoV5-1, 40CrMnMo7 and 90MnCrV8. The investigated tool steels were heat-treated, while K340 was subjected to thermochemical treatment and then coated with an AlCrSiN hard film (K340/AlCrSiN). The hardness, chemical composition, phase structure and microstructure of steels K340 and K340/AlCrSiN were examined. Tribological tests were conducted using the ball-on-disc tester in compliance with the ASTM G99 standard. The tests were performed under dry unidirectional sliding conditions, using an Al_2_O_3_ ball as a counterbody. The wear factor and coefficient of friction were estimated and analyzed with respect to hardness and alloying composition of the materials under study. Scanning electron microscopy (SEM) observations were made to identify the sliding wear mechanisms of the analyzed tool steels and physical vapor deposition (PVD)- coated K340 steel. In contrast to the harsh abrasive–adhesive wear mechanism observed for uncoated tool steels, the abrasive wear dominates in case of the AlCrSiN. The deposited thin film effectively prevents the K340 substrate from harsh wear severe degradation. Moreover, thanks to the deposited coating, the K340/AlCrSiN sample has a coefficient of friction (COF) of 0.529 and a wear factor of *K* = 5.68 × 10^−7^ m^3^ N^−1^ m^−1^, while the COF of the reference tool steels ranges from 0.70 to 0.89 and their wear factor ranges from 1.68 × 10^−5^ to 3.67 × 10^−5^ m^3^ N^−1^ m^−1^. The AlCrSiN deposition reduces the wear of the K340 steel and improves its sliding properties, which makes it a promising method for prolonging the service life of metalwork tools.

## 1. Introduction

Despite the development of new technologies such as sintered carbides and ceramic materials, tool steels are still widely used in the industry. This results from the high quality, wide availability and fair price of these materials. To obtain an effective and durable tool, it is necessary to employ a suitable heat treatment or surface layer modification technology or to deposit a coating with the required properties. Another important aspect is to select a steel grade that exhibits properties meeting the requirements for a given tool application and maximizes the tool durability during the cold- or hot-working [1,2,3]. Studies are still conducted to develop new materials [4] and methods for shaping microstructure, particularly surface layer, of tool steels [5].

Tool steels are characterized by high hardness and resistance to abrasion and deformation, as well as the ability to withstand elevated temperatures. These characteristics can be obtained by increasing the carbon content and the application of appropriate heat treatment as well as the use of high alloy steel grades and heat treatment, or by the application of appropriate coatings [6,7].

A key requirement for cold-work carbon steels is their high hardness and resistance to abrasion. If high surface pressures are generated during tool operation, it is necessary to increase the resistance of core and surface layer or apply the coating. The surface layer resistance must be high enough to carry the tool operation load and stresses. This can be obtained by enriching steel with alloying elements. The most popular grades of tool steel contain alloying elements showing a close affinity with carbon, i.e., Cr, Mo, W and V, which means that hard phases can be formed in the microstructure of these steels. Such microstructure of steel leads to increased resistance to abrasion. The use of alloying elements in appropriate proportions makes it possible to optimize strength and abrasive properties of tools [5,8].

A current trend is to improve the tribological performance of cold work tools. Different attempts are made to this end, including transition-metal nitride coatings of (Ti,Zr)N [9], for which the smallest wear rate was obtained at the atomic percentage of 46% nitrogen. The authors of [10] investigated the characteristics of nitrogen atmosphere heat-treated CrAlSiN films after physical vapor deposition (PVD) coating and found that the PVD CrAlSiN film was able to retain the initial structure after annealing up to 800 °C. The wear resistance of a PVD-TiAlN-coated tool was found superior compared to that of a fine shot peening modified surface tool [11]. Following the results, many authors use PVD TiN-based thin films to reduce wear factor and mitigate the degradation processes of metallic substrates [12,13].

The major advantages of PVD include the following: almost unlimited variation regarding the chemical composition of the coating material, the principal tolerance of all substrate materials and the possibility of depositing compounds such as nitrides and carbides or materials such as diamond-like carbon. The advantages of PVD also include the easy realization of layered or graded film structures. Apart from the advantages, the technology also has some drawbacks, including the necessity of using vacuum and plasma equipment together with the line-of-sight process type, as well as the complex sample movement. A high cost of PVD deposition in comparison with other technologies may be a significant disadvantage in the case of some applications. PVD requires the use of complex machines (which are more and more widely available) and thus skilled operators. In addition, the application of PVD coating is not the best solution for limited-time treatments because it usually takes a longer time than other methods.

Some researchers attempt to increase abrasion resistance of steels by depositing different combinations of coatings [9,14,15] (also known as nanolayers [16]), including those with only slightly different chemical composition [17,18,19] or chemical-composition gradient [20,21]. The highest improvement of the double layered CrAlSiN + CrN/C coating was nearly three times more reliable than that of the tool coated with a single CrAlSiN layer [22]. According to Li [23], the elastic modulus is a linear function of hardness (in GPa units) of nitride coatings, but the elements which do not create nitrides (Co, Cu, Fe, Ni and Mn) are inclined to form a metallic phase, which can disturb the crystalline structure and lead to amorphization.

Nitriding and/or PVD coating are popular surface treatment processes for hot-work tool steels. The nitriding process helps increase the hardness of a workpiece surface up to at least 1200 HV. Therefore, hardness of PVD deposited nitride binary coating systems does not exceed 30 GPa [24] which can be achieved by deposition of complex coating systems such as AlCrN and AlCrSiN [17,25]. The high functional properties of nitride coatings deposited on alloy steels result from the fact that such coatings remain stable at elevated temperature [26]. Given that this treatment is characterized by a lower temperature than the polymorphic transition temperature and a longer time, PVD coatings can be deposited after quenching, sometimes together with tempering. Nitride PVD coatings increase the potential applications for steels because—apart from high resistance to surface stresses/loads and abrasion—they also ensure resistance to the harsh environment. The deposition of the Al-rich AlTiN thin films successfully prevents the steel substrate from the sliding wear and cavitation erosion [27], while the silicon enrichment of the CrAlN coatings increases hardness by approximately 34% [28] and salt water resistance [29]. The resistance to oxidation at elevated temperatures induces the formation of Al, Cr and Si oxides that remain stable up to 700 °C [28]. The CrAlSiN coating deposited by unbalanced magnetron sputtering exhibits better tribological and mechanical properties up to 700 °C than the CrAlN coating, according to nanoindentation and tribological tests [30,31]. In addition, the average wear factor (about 0.08) of the CrAlSiN coating was lower at high temperature [32]. CrAlSiN hard coatings with a metastable cubic wurtzite structure, where Al substitutes Cr in the CrN-based structure, are used for manufacturing dies, molds and cutting tools due to their properties, primarily high wear and oxidation resistance [19,25,33].

Despite many tribological studies on PVD-coated tool steels, according to the authors knowledge, none of the recently published papers compares the wear behavior of AlCrSiN coating with a set of popular tool steels including grade K340. The results of the anti-wear investigations are still important and interesting for scientists and technologists. Therefore, this paper presents results of tribological studies on lesser-known cold-work steel K340. The literature review demonstrates that the K340 steel has not been exhaustively tested. There is practically no description of its tribological performance in the literature. Compared to tool steels, this steel grade should offer many advantages, such as good machinability and small dimensional changes during heat treatment [34]. The K340-coated by the hard films could broaden the steel future applications at elevated temperatures. This research compared the quantitative wear resistance of AlCrSiN PVD-coated steel grade K340 with the bare steel K340 and a set of tool steels, as this problem has not been yet well documented in the literature. Additionally, the current paper presents an innovative comparison of the sliding wear results obtained for K340 and a set of popular tool steels designated for cold- and hot-working, which gives an interesting remark for tool steels selection and performance. Moreover, the dry sliding wear behavior and damage mechanisms of the K340 steel coated with an AlCrSiN thin film were investigated in relation to those of the reference tool steels. The results presented in this paper may prove useful for fabrication and prolonging the service life of cold-working tools and this research introduces to the broader project for investigating the durability of the K340-coated tool steel at elevated temperatures.

## 2. Materials and Methods

The main goal of this study was to investigate the differences in quantitative wear behavior and wear mechanism between AlCrSiN-coated and bare K340 steel and five reference tool steels: X155CrVMo12-1, X37CrMoV5-1, X40CrMoV5-1, 40CrMnMo7 and 90MnCrV8. There are several reasons for selecting PVD-coated and uncoated K340 as well as cold- and hot-work tool steels as materials for analysis. First, the wear behavior of steel K340 has not yet been comprehensively described in the scientific literature. In addition, there are no studies describing the effects of depositing AlCrSiN coatings onto the K340 steel substrate, which is the primary objective of our study. This coating is a universal candidate for both cold and hot metal forming and advanced cutting tools. The AlCrSiN film deposition could facilitate the steel K340 operation at both room and elevated temperatures. Secondly, to ensure a comprehensive analysis of the tribological performance of the K340 steel, we selected popular cold- and hot-working tool steel grades as reference materials. Thirdly, while selecting the reference materials, we took into account the chemical composition of steel K340. The chemical composition of K340 ranges in between the chemical element contents of the reference tool steels. Finally, the literature does not provide any data about the wear rate of AlCrSiN thin films in comparison to the K340 tool steel. Therefore, this work undertakes a quantitative comparison of tribological behaviors and wear rates for the coated steel and the references steel grades.

Two types of the K340 samples were investigated in the study, denoted as K340 (heat-treated) and K340/AlCrSiN (heat treated and PVD coated). The steel grade K340 is used for cold work tools. Its average hardness, measured according to standard [35], in as-received condition was 225 HBW. The K340 samples were first subjected to austenitization in a vacuum furnace and then quenched with an N_2_ (5 bar) string (see Table 1). The final obtained hardness of the samples was equal to 62 HRC [36]. After quenching, the K340 steel samples were subjected to four tempering processes. Three of them involved heating the samples for 120 min to the temperature of 505 °C and soaking for 240 (first tempering process) or 210 min (the next two processes). In the final tempering process, the samples were heated up to 510 °C for 120 min and soaked for 240 min. Following every tempering process, the material was air-cooled.

One batch of K340 steel samples was prepared for further testing. The other batch was first heat-treated and then subjected to additional treatment (nitriding and PVD). In accordance with the objective of this study, the tribological behavior of the K340 and K340/AlCrSiN samples was analyzed in relation to the hardness and wear properties of popular hot- and cold-work tool steels. For comparison purposes, two hot-work tool steel grades (X37CrMoV5-1 and X40CrMoV5-1) and three grades of cold-work steel were selected. Standard chemical compositions of the tested tool steels are given in Table 2. Data in the table are shown in a chromium content descending order. All analyzed steel samples were subjected to quenching and tempering heat treatment. Additionally, the chemical analysis of the K340 steel was performed with the Magellan Q8 spark emission spectrometer (Bruker, Germany); the Fe100 test channel was used to complete five analyses (sparking sequences) for every sample.

The samples used for hardness, chemical composition and tribological testing were made as discs with a diameter of ø25 mm and a thickness of 6 mm. The steel discs were subjected to grinding with water abrasive papers with the grain size of 200, 400, 600 and 1200, respectively. After grinding, the samples were mechanically polished with a 3 μm diamond particle suspension and 0.05 μm oxide particle suspension, washed in acetone and dried. Microstructures of the K340 and K340/AlCrSiN samples were examined by bright-field optical microscopy using Nikon MA200 (Nikon Corporation, Tokyo, Japan) and scanning electron microscopy with energy dispersive spectroscopy (SEM-EDS, Phenom World ProX, Phenom World, Waltham, MA, USA). The chemical composition of AlCrSiN thin film was analyzed in the cross-section of the samples by SEM-EDS method.

Phase composition of the samples was identified with the use of the X-ray diffractometer (XRD) model ARL X’tra from Thermo Fisher. A filtered copper lamp (CuKα, λ=0.1542 nm), with a voltage of 40 kV, range $2\theta =20^{\circ }-120^{\circ }$ and step size 0.02${}^{\circ }$/3 s was used. Phase composition was determined using the powder diffraction file (PDF) developed and issued by the International Centre for Diffraction Data (ICDD).

Hardness tests were conducted using the Vickers FM-700 microhardness meter with an automatic ARS 900 system (Future-Tech Corp.), according to standard [37]. To ensure statistical accuracy, at least seven indentations were made in random locations. After that, the Rockwell hardness was recalculated into the Vickers scale in compliance with the ISO 18265 standard [38]. The deposited nitride film hardness was tested on the top of AlCrSiN film surface using an Ultra Nanoindentation Tester (Anton Paar GmbH, Ostfildern, Germany), in compliance with the procedures described in [39]. The thin film nanohardness was measured for comparison with the surface macro hardness measured with Vickers hardness tester.

Wear tests were performed on a “ball-on-disc” tribotester manufactured by CSM Instruments. Al_2_O_3_ balls (manufactured by CSM Instruments) with a diameter of 6 mm were used as counterbodies. The total test distance used for measuring coefficient of friction (COF) variation for a single sample was set equal to 1000 m. The tests were performed at room temperature, under the conditions described in Table 3.

Wear was measured as the reduction of material volume in the form of a wear track resulting from the specimen–counterbody interaction. The Dektak 150 profile contact tester from Veeco Instruments was used to measure the wear profile surface area along the specimen circumference (in 12 locations). The wear volume was determined as the average value wear profile areas and the circumference of a wear track circle created during the ball-on-disc test. After that, the wear factor *K* was determined by Equation (1) considering the wear volume, force and sliding distance in the test:

(1) $$K=\frac{Wear\;volume}{Applied\;force\times Sliding\;distance}$$

for which the unit is $mm^{3}\;N^{-1}\;m^{-1}$. After the tribological tests, to investigate the sliding wear mechanism, the samples wear tracks were examined using scanning electron microscope.

## 3. Results and Discussion

### 3.1. Materials Properties

Results of the hardness tests are given in Table 4. Data in the table are ordered by Vickers hardness. Following the heat treatment, the surface layer hardness of steel K340 is considerably higher (by at least 78 HV) than that of other tested steel grades. As a result of the applied heat treating processes, i.e., nitriding and AlCrSiN coating deposition, the hardness of this material (samples denoted as K340/AlCrSiN) increased again by almost two times to over 1300 HV. Regarding the five other tested grades of steel, their post-treatment mean hardness ranges between 518n and 669 HV. These five steel grades include two heat-treated hot-work steels with a hardness of approximately 550 HV. Moreover, the AlCrSiN hard film hardness estimated with nanohardness tester equals 29.1 ± 8.9 GPa, which is superior to the hardness results obtained with the Vickers tester. The estimated nano-hardness is in the range of values reported in the literature [40] and it agrees with the hardness reported for other nitride hard-films such us TiAlN, AlTiN and AlCrN [31,41].

**Figure 1:**
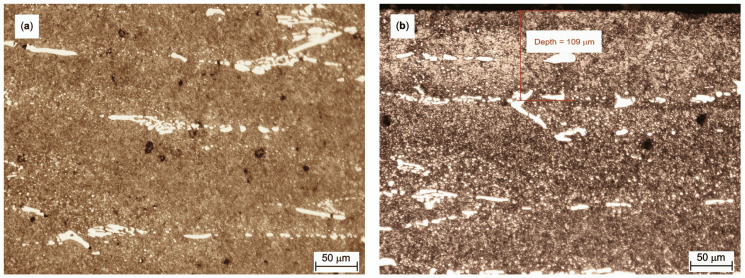
Microstructure of the tested K340 steel samples: (**a**) core-area; and (**b**) nitrided surface layer.

Microstructure of the heat-treated K340 steel is shown in Figure 1. Despite the presence of spheroidized carbides, martensite lathes are visible in the structure of the K340 sample. The diffusion nitride layer on the K340 steel sample is visible to an average depth of about 100 μm below the surface (Figure 1b). One can see a two-phase structure with the sorbite matrix and brighter carbides (Figure 1a). In addition, two types of carbides can be distinguished. One is characterized by elongated grains of ferrite-based solid solution that are shredded relative to the direction of plastic deformation which took place during steel fabrication. The other contains more uniformly distributed fine precipitations of alloy carbides (up to a few μm in size) that, according to the literature of the subject [42], are typically generated during tempering. The microstructure in the center of the sample is similar to the structure shown in Figure 1b.

**Figure 2:**
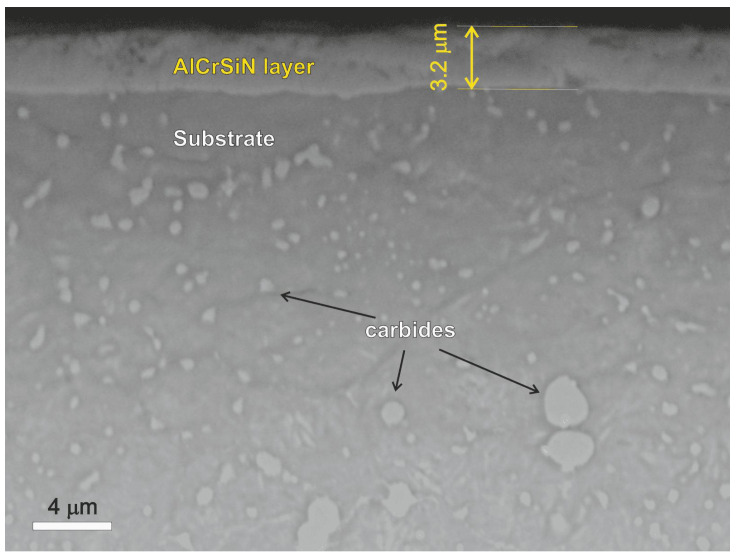
Scanning electron microscopy (SEM) image of K340/AlCrSiN sample.

Figure 2 shows the SEM image of the AlCrSiN coating cross section on the nitride layer in the K340/AlCrSiN sample. The thickness of PVD coating on the K340/AlCrSiN sample varies from 2.8 to 3.6 μm. Table 5 gives the average chemical composition of the deposited AlCrSiN coating obtained from EDS measurements for seven spots. Data are ordered by atomic concentration of particular elements. It can be seen that the coating has more Al atoms than Cr. Nevertheless, the coating has an atomic stoichiometry of Al${}_{0.23}$Cr${}_{0.17}$Si${}_{0.03}$N, which is comparable with data reported in the literature [43,44,45].

**Figure 3:**
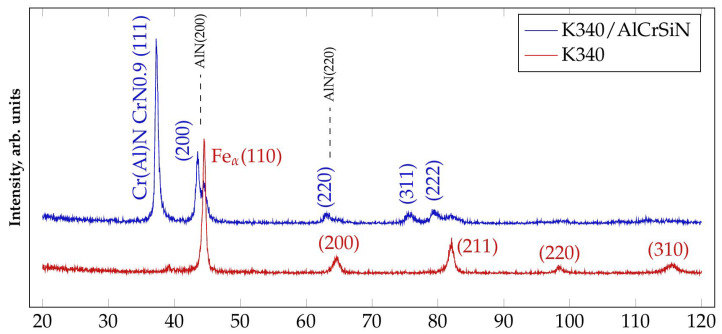
X-ray diffraction (XRD) pattern of the K340 sample in as-received condition and after AlCrSiN coating deposition.

X-ray diffraction (XRD) was employed to analyze the phase content of steel K340 in as-received condition and after AlCrSiN coating deposition by PVD. The obtained results are given in Figure 3. The sample of K340 in as-received condition contains a ferrite phase (ICDD 04-002-1833) and neither cementite nor other carbides were identified by XRD, which is a typical result supported by the literature [46]. Moreover, the presence of carbides is clear and can be clearly seen in Figure 1 and Figure 2. Nevertheless, the phase composition of the nitride layer significantly differs from that of the K340 substrate. Following the coating deposition, two phases can be distinguished: Cr(Al)N (ICDD 04-021-7700) and CrN0.9 (ICDD 01-083-5613). The peaks of the CrN and AlN phases nearly overlap, being pairs with identical lattice planes (Miller indices), and are thus hard to differentiate. Summing up, the structure of the thin film consists on the diverse crystalline phases and agrees with the XRD of AlCrSiN coatings reported by the literature [17,25].

### 3.2. Wear Behavior

**Figure 4:**
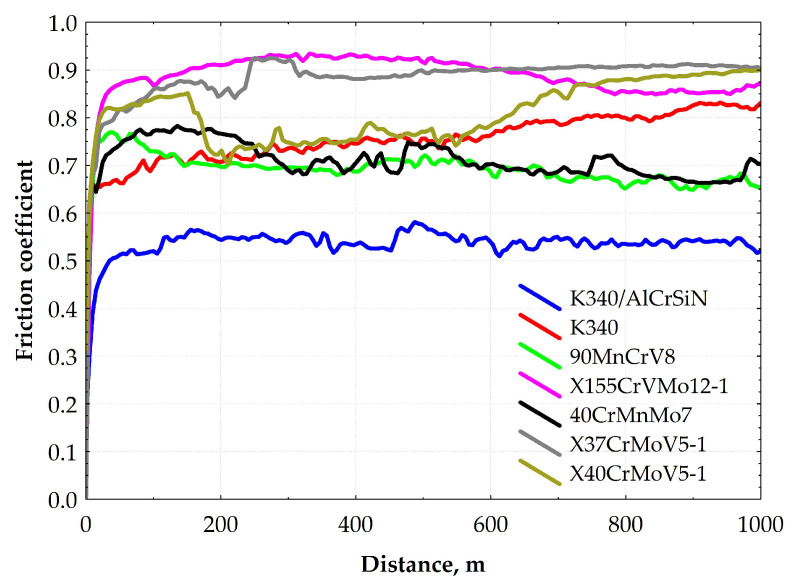
Curves illustrating variations in friction coefficient vs. distance for the tested materials.

**Figure 5:**
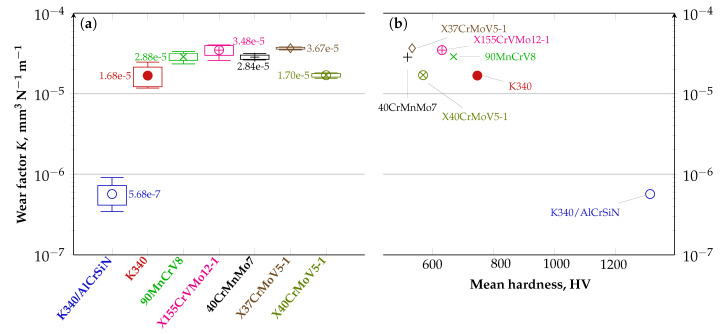
Diagram illustrating the wear factor *K*: (**a**) for the tested materials; and (**b**) as a function of surface hardness.

**Figure 6:**
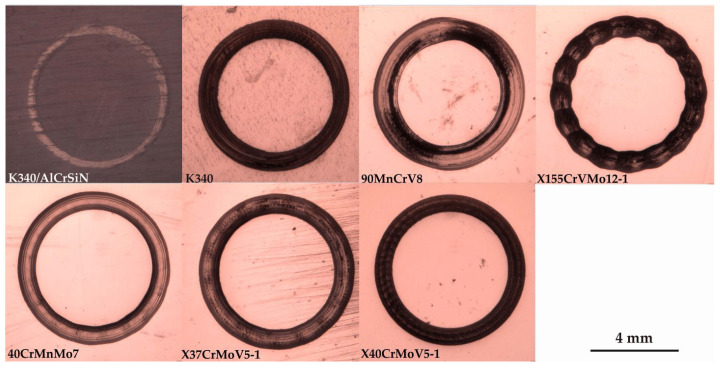
Worn surface of the samples tested under dry friction conditions.

A comparative analysis was conducted on the basis of the dry sliding ball-on-disc test done for all samples under the same environmental conditions. Obtained average coefficients of friction are given in Table 6 and plotted in relation to distance in Figure 4. The data in the table are listed in a reverse order of average friction coefficients. For most tested steels, the average COF ranges between 0.70 and 0.89. Regarding the cold-work steels, the highest COF was achieved by the X155CrVMo12-1 steel grade that is characterized by the highest carbon and chromium content out of the analyzed materials. As for the other tested cold-work steel grades, the COF variations are not statistically significant considering the standard deviation. It should, however, be pointed out that the lowest average COF (with narrow range variations over distance) is obtained for the K340/AlCrSiN sample. The deposition of the AlCrSiN thin film onto the nitrided surface layer of the K340 steel sample effectively decreases the COF to an average value of 0.53. The COF of the K340/AlCrSiN sample is lower than that of the steel samples due to superior hardness of the AlCrSiN film. This implies that the abrasive wear mechanism is dominant in the thin film wear trace (as discussed in detail in a further section of the paper), resulting in a low wear volume (Figure 5 and Figure 6) and stable wear process.

During the tribological test, the COF of most samples is stable and the standard deviation is lower by at least one order of magnitude than the average value (Table 6). The highest COF variation (standard deviation amounting to 12% of the average value) is observed for the sample of quenched and tempered K340. This unstable behavior pattern of the COF may be explained by the microstructure of steel K340, in particular the carbide banding. The numerous and relatively large carbides (Figure 1a and Figure 2) constitute a natural obstacle for the counterbody material. After the wear-in stage, the contact surface area between the mating surfaces is increased and, in turn, the COF fluctuations are reduced. As for the hot-work steels tested under dry friction conditions, both grades end up with the sliding distance with the relatively high COF amounting to 0.9, which exceeds the values reported for cold work steels.

Figure 5 illustrates the wear factor *K* of the tested materials. It can be read that the wear resistance of K340/AlCrSiN is almost two orders of magnitude higher than that of all reference materials. The results in this figure also reveal that the wear resistance of steel K340 is in the range of the wear rate reported for the steel grade X40CrMoV5-1. The AlCrSiN thin film deposition on the K340 steel (sample denoted as K340/AlCrSiN) leads to a further significant increase in the wear resistance of this sample compared to the other tested steels hot- and cold-work alike.

Figure 6 shows the macro-photographs of worn surfaces. It can be observed that, apart from the K340/AlCrSiN sample, all other tool steels are characterized by the adhesive-smearing mechanism in the wear track. In the case of the four steel grades with high values of wear factor *K*, i.e., 90MnCrV8, X155CrVMo12-1, 40CrMnMo7 and X37CrMoV5-1, the outside edges of the tracks additionally exhibit plowing of the wear products. This points to galling and cyclic upsetting of the high portion of the deformed tribofilm. This effect is the most noticeable in the wear track obtained for the X155CrVMo12-1 steel with the lowest wear resistance. A similar material-deterioration morphology in the wear track, characterized by the dominant adhesive wear mode, was also observed for untreated Al- and Cu-based metal alloys tested at ambient temperature under dry sliding conditions [47].

Table 7 shows the mean depth of obtained wear tracks. The observed fluctuations in the profile depth can be explained by non-uniform deterioration of the wear track in a given wear mechanism. The wear volume can be positive because of the adhesive wear mode. To give an example, the wear mechanism of the X37CrMoV5-1 and X40CrMoV5-1 samples relies on an adhesive transfer of the worn material through the wear track and a relatively low penetration depth (Table 7).

**Figure 7:**
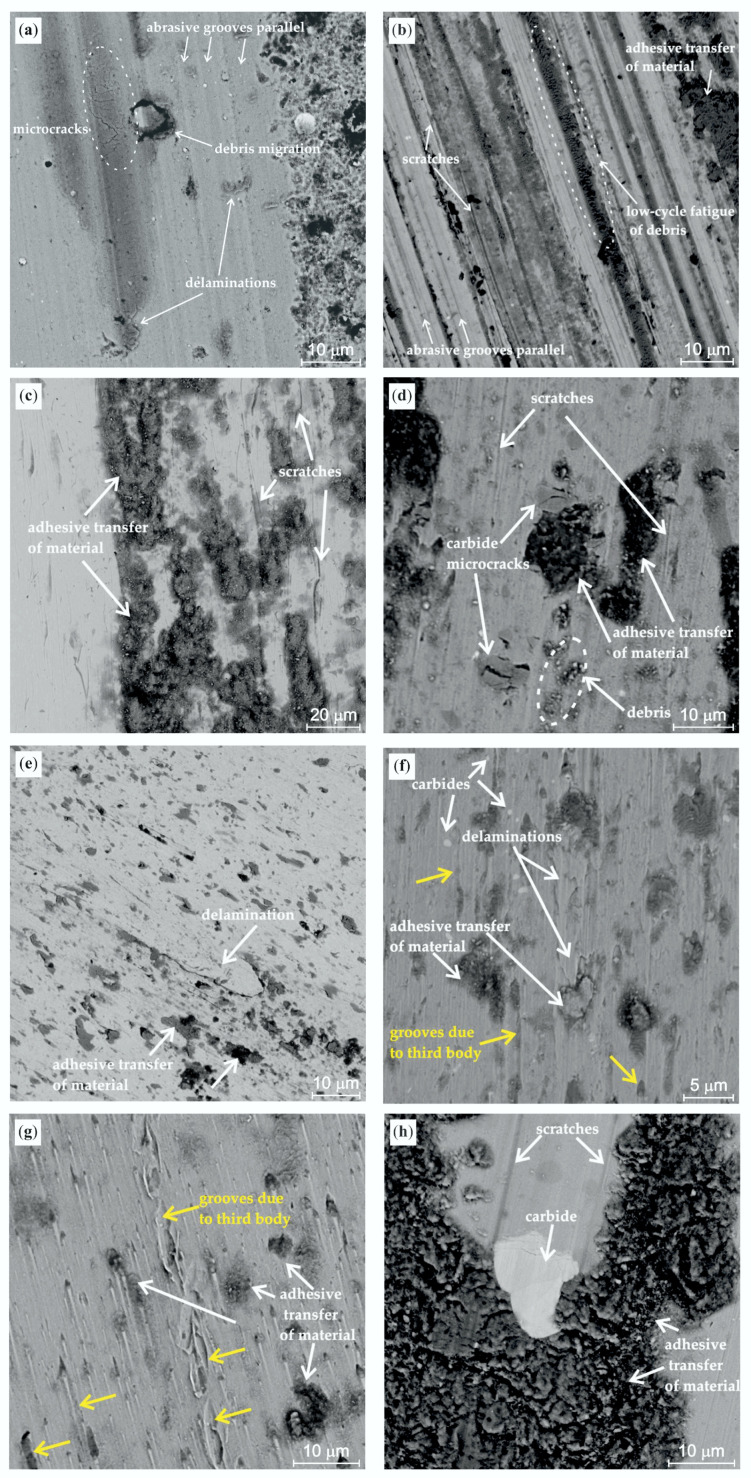
SEM microphotographs of the worn surfaces of: (**a**) K340/AlCrSiN; (**b**) K340; (**c**) 90MnCrV8; (**d**) X155CrVMo12-1; (**e**) 40CrMnMo7; (**f**) X37CrMoV5-1; (**g**); and X40CrMoV5-1 (**h**).

SEM examination of the obtained wear tracks made it possible to identify dominant wear mechanisms (Figure 7). The results of SEM examination agree with the quantitative wear results. The reason for the high wear resistance of the PVD-coated sample is the fact that the wear mechanisms of the thin film and tool steels differ. The high wear resistance of the PVD-coated sample can be attributed to the dominance of abrasive wear, whereas the steels predominantly undergo adhesive wear. This results in higher wear rates and friction coefficients of all tested tool steels. It is known from the literature [48] that adhesive wear results in a lower wear rate of samples than the abrasive wear mode.

The K340/AlCrSiN sample (Figure 7a) exhibits the abrasive nature of wear. The cyclic sliding process causes film cracking and fatigue-inducted delamination followed by a transfer of the spalling debris material across the wear track. Additionally, the nitride coating shows the presence of lateral microcracks, which is the symptom of fatigue. On the other hand, the wear of tool steels is affected by the adhesive damage and its mechanism has an abrasive–adhesive nature. In the case of the K340 sample (Figure 7b), the wear process primarily starts with abrasion and proceeds in an abrasive–adhesive mode. The worn surface of the K340 sample shows the presence of characteristic parallel abrasive grooves and scratches caused by microcutting. In addition, the grooves have microcracks propagating perpendicularly to the sample movement direction. These microcracks result from low-cycle fatigue of the surface layer due to the cyclic upsetting of the material. At the same time, adhesive wear occurs, as demonstrated by the presence of the smeared tribofilm resulting from the transfer of secondary wear products.

The wear track surface of the 90MnCrV8 and X155CrVMo12-1 steels (Figure 7c,d) reveals the presence of continuous scratches, which is typical of steel and is caused by the free displacement of wear products along the sample/counterbody contact trace. Moreover, as in the case of steel K340, these steels grades also, simultaneously, undergo adhesive wear, which is particularly visible for 90MnCrV8 (Figure 7c). Generally, two patterns of material adhesive transfer can be observed for all tested reference steel samples: the first relies on delamination of the initial metallic material, while the second consists in wear debris transfer and final smearing.

Krbata et al. [49] demonstrated that the adhesive wear mechanism of this steel grade is more intensive at higher velocities during tribotesting. In turn, steel X155CrVMo12-1 exhibits the microcracking of carbides, which was probably caused by surface deformation of the material (Figure 7d). The carbide microcracks are the center of fracture nucleation as well as fatigue spalling and may lead to tool damage, as demonstrated in [50].

An analysis of the wear surface of the 40CrMnMo7 sample (Figure 7e) shows the presence of abrasive wear (bright areas) and delamination together with the adhesive transfer of wear products along the wear path (dark areas). The wear surface of the tested X37CrMoV5-1 and X40CrMoV5-1 steel samples is similar (Figure 7f,g) with the exception of X37CrMoV5-1 where delamination can additionally be observed on the wear track surface. The susceptibility to delamination of this steel grade can be explained by a relatively low hardness. As a result, this material is prone to this kind of plastic deformation. In addition, due to a high content of chromium and carbon, the microstructure of X40CrMoV5-1 contains a higher amount of hard phase [51], which could lead to a higher wear resistance than that of the X37CrMoV5-1 sample in the ball-on-disc test. In the case of X40CrMoV5-1 steel (Figure 7g,h), the wear process was intensified by the additional abrasive action of wear debris that moving freely between the sample and counterbody surfaces and were rammed into the wear track surface.

## 4. Conclusions

The knowledge of tribological characteristics and wear mechanisms of materials makes it possible to develop comprehensive criteria of their selection when designing products for tool steels in the manufacturing industry. The main goal of this study was to investigate the differences in quantitative wear behaviors and wear mechanisms between the AlCrSiN-coated and bare steel K340 and the reference tool steels X155CrVMo12-1, X37CrMoV5-1, X40CrMoV5-1, 40CrMnMo7 and 90MnCrV8. The results presented in this study may prove useful for manufacturing cold-working tools and prolonging their service life.

The results of the ball-on-disc test conducted under dry sliding conditions have confirmed that the PVD AlCrSiN coat deposited onto the nitrided surface of steel K340 reduces the wear of this steel grade and improves its sliding properties. Therefore, the lowest COF of 0.53 and the wear factor of *K* = 5.68 × 10 ^−7^ mm^3^ N^−1^ m^−1^ were reported for the PVD-coated sample. The superior wear resistance of the K340/AlCrSiN sample results from its higher hardness than those reported for steels, leading to the dominance of abrasive wear. Additionally, the nitride coating shows the presence of lateral microcracks, which is the symptom of simultaneously occurring fatigue-induced thin film spallation and material transfer.

Regarding the tool steel samples, the highest wear resistance (estimated by average wear rate K) is observed for the samples in the following order: K340 > X40CrMoV5-1 > 40CrMnMo7 > 90MnCrV8 > X155CrVMo12-1 > X37CrMoV5-1. Their COF ranges from 0.70 to 0.89 and the wear factor ranges from 1.68 × 10^−5^ to 3.67 × 10^−5^ mm ^3^ N^−1^ m^−1^. This is related to the presence of hard carbide phases embedded in the ferrous matrix in their microstructure. This implies that the sliding wear behavior is abrasive–adhesive in nature. Following microcutting, parallel abrasive grooves are visible on the wear track surface; it is also determined that the adhesive transfer of the material relies on two patterns: the first one is the delamination of the initial metallic material, while the other consists in the transfer and final smearing of wear debris.

Summing up, in comparison to the set of reference tool steels, the PVD-deposition of AlCrSiN onto K340 steel reduces the wear rate and coefficient of friction, consequently improving its sliding properties. Contrary to the abrasive–adhesive behavior of tool steels, the wear mechanism of K340/AlCrSiN has the abrasive mode and, therefore, successfully decreases the material loss. Application of AlCrSiN coating seems promising method for prolonging the service life of metalwork and cutting tools manufactured from K340 steel.

## Figures and Tables

**Table 1:** Technological parameters of heat treatment of the K340/AlCrSiN samples.

Stage Chronologically		Time, min	Stage Temperature, °C		
			at the Beginning	Continuous	at the End
Austenitization	preheating	210	ambient		700
	soaking	180		700	
	reheating	60	700		870
	soaking	180		870	
	reheating	45	870		1030
	soaking	90		1030	
	N${}_{2}$ quenching		1030		ambient
Tempering	preheating	120	ambient		505
	tempering I	240		505	
	air cooling		505		ambient
	preheating	120	ambient		505
	tempering II	210		505	
	air cooling		505		ambient
	preheating	120	ambient		505
	tempering III	210		505	
	air cooling		505		ambient
	preheating	120	ambient		510
	tempering IV	240		510	
	air cooling		510		ambient
Nitriding	preheating	130	ambient		450
	soaking	240		450	
	reheating	130	450		540
	nitriding	2400		540	
	air cooling		540		ambient
	PVD ^1^	300		445	

^1^ Physical vapor deposition.

**Table 2:** Chemical composition of the tested K340 steel and the set of reference tool steels.

Steel Grade	Content of Element (Fe—Balance), wt. %									
	C	Si	Mn	Cr	Mo	Ni	V	W	S	P
X155CrVMo12-1	1.525	0.350	0.400	12.000	1.000		0.850		0.030	0.030
K340	1.100	0.900	0.400	8.300	2.100		0.500			
K340 ^1^	1.111	0.767	0.406	8.297	1.961	0.329	0.530	0.071	0.002	0.021
X40CrMoV5-1	0.385	1.000	0.375	5.150	1.350		1.000		0.030	0.020
X37CrMoV5-1	0.370	1.000	0.375	5.150	1.300		0.400		0.030	0.020
40CrMnMo7	0.400	0.300	1.450	1.950	0.200				0.030	0.030
90MnCrV8	0.900	0.250	2.000	0.350			0.125		0.030	0.030

^1^ Results of spectrometer analysis.

**Table 3:** Parameters of tribological tests.

Parameter	Load	Linear Speed	Rotational Diameter
Unit	N	cm s^−1^	mm
Value	10	10	6

**Table 4:** Treatment parameters and overall surface hardness of the tested materials.

Steel Grade	Work	Processing Temperature, °C			Hardness			
		Austenitizing	Tempering	PVD	HRC	SD ^1^, HRC	HV	SD ^1^, HV
K340/AlCrSiN	cold	1030	505 ÷ 510	445			1314	92
K340	cold	1030	505 ÷ 510		62.0	1.2	747	25
90MnCrV8	cold	1800	270		58.8	0.6	669	12
X155CrVMo12-1	cold	1020	270		57.0	0.2	631	5
X40CrMoV5-1	hot	1000	300		53.5	0.9	569	14
X37CrMoV5-1	hot	1020	270		51.3	0.9	533	15
40CrMnMo7	cold	800	270		50.3	0.8	518	12

^1^ Standard deviation.

**Table 5:** Average contents of elements in the analyzed AlCrSiN layer.

Symbol	Concentration			
	Atomic, at. %	SD, at. %	Weight, wt. %	SD, wt. %
N	57.0	4.0	33.4	2.3
Al	23.2	1.3	26.1	1.5
Cr	17.3	4.1	37.4	8.8
Si	2.6	0.1	3.0	0.2

**Table 6:** Friction coefficients of the tested samples.

Sample Steel	Work	Average Friction Coefficient	Standard Deviation
X37CrMoV5-1	hot	0.89	0.06
X155CrVMo12-1	cold	0.88	0.07
X40CrMoV5-1	hot	0.81	0.06
K340	cold	0.74	0.09
40CrMnMo7	cold	0.71	0.04
90MnCrV8	cold	0.70	0.05
K340/AlCrSiN	cold	0.53	0.05

**Table 7:** Comparison of wear trace depths obtained for the tested samples.

Sample	Wear Trace Depth, μm	Standard Deviation, μm
K340/AlCrSiN	21.64	0.38
K340	16.02	2.70
90MnCrV8	20.94	1.79
X40CrMoV5-1	21.07	1.86
X37CrMoV5-1	21.54	0.83
40CrMnMo7	27.42	1.39
X155CrMoV12-1	29.34	3.01
